# A Scoping Review of Integrated Care Models for Managing Depression in Older Adults

**DOI:** 10.3390/geriatrics11050126

**Published:** 2026-09-08

**Authors:** Mia Haddad, Jittima Panyasarawut, Chaowalit Srisoem, Dennis Miezah, Ling Shi

**Affiliations:** 1 Department of Nursing, Manning College of Nursing and Health Sciences, University of Massachusetts Boston, Boston, MA 02125, USA; mia.picillo001@umb.edu (M.H.); j.panyasarawut001@umb.edu (J.P.); chaowalit.srisoem001@umb.edu (C.S.); dennis.miezah001@umb.edu (D.M.); 2 Department of Adult and Geriatric Medicine, Massachusetts General Hospital, Boston, MA 02114, USA; 3 Department of Fundamental Nursing, Faculty of Nursing, Mahidol University, Nakhon Pathom 73170, Thailand; 4 Department of Psychiatric and Mental Health Nursing, Boromarajonani College of Nursing Sunpasitthiprasong, Faculty of Nursing, Praboromarajchanok Institute, Ubon Ratchathani 34000, Thailand

**Keywords:** scoping review, integrated care model, depression management, older adults

## Abstract

**Background:** Depression is a major health issue among older adults and is frequently accompanied by chronic physical conditions. Conventional care of depression in older adults is delivered through primary care settings and often fails to address the complex interactions between mental health, physical illness, and social determinants of health in this population. Integrated care models, characterized by coordinated, multidisciplinary, and patient-centered approaches, have emerged as a promising strategy for improving depression outcomes in late life. **Objective:** This scoping review aimed to characterize integrated care models for managing depression in older adults. The review examined the structure and implementation of these models and evaluated depression-related outcomes. **Methods:** A systematic literature search was conducted in PubMed and Google Scholar. Studies published in English between 2006 and February 2026 were considered. Eligible studies included randomized controlled trials (RCTs), cluster RCTs, and quasi-RCTs that evaluated integrated care interventions targeting depression in adults aged 65 years or older or mixed adult populations that included older adults. One reviewer conducted the search and initial screening, and four additional reviewers evaluated the eligibility of full-text reports and extracted data using a structured template. Results were synthesized descriptively. **Results:** Seventeen studies met the inclusion criteria. Integrated care interventions were implemented across primary care clinics, community health centers, and home healthcare programs in several countries. Participants commonly had depression alongside chronic medical conditions, including diabetes, hypertension, heart failure, or chronic obstructive pulmonary disease. Integrated care models varied in staffing, therapeutic components, specialist involvement, delivery intensity, and comparator conditions. Most interventions incorporated multidisciplinary care teams; structured symptom monitoring; care coordination; behavioral or psychological interventions; and, in some studies, chronic disease management, social support, or technology-supported follow-up. Thirteen studies reported a favorable depression-related finding at one or more assessment points; however, benefits were not always sustained, and some studies reported null- or comparator-favoring findings. **Conclusions:** Integrated care for depression in older adults encompasses heterogeneous models implemented across diverse healthcare contexts. Common themes included multidisciplinary collaboration, coordinated follow-up, structured psychological or behavioral treatment, symptom monitoring, and integration of mental and physical health management. Favorable outcomes were reported in most studies, but findings varied by population, setting, comparator, intervention intensity, and follow-up duration. These findings may inform future program design, although the effectiveness of the integrated care models and the independent contribution of individual components remain uncertain.

## 1. Introduction/Background

Depression is one of the most common and disabling mental health disorders among older adults, affecting approximately 10% to 20% of this population globally [1]. It is associated with functional impairment, reduced quality of life, and increased mortality. Given the rapidly aging population worldwide, the global burden of late-life depression is significant and represents a significant public health concern. Older adults frequently experience depression alongside chronic medical conditions such as diabetes, hypertension, cardiovascular disease, and chronic obstructive pulmonary disease [2,3]. These comorbidities complicate both diagnosis and treatment, making management more complex and often requiring coordination across multiple healthcare domains.

Despite the growing burden, traditional approaches to depression treatment typically begin in primary care settings, with referral to specialized mental health services when needed [4]. Depending on the healthcare system, usual care may include depression assessment and management by primary care clinicians, the prescription of antidepressants, general follow-up, chronic disease management, or referral to mental health services. While the current model provides an entry point for care, it often fails to address the complex and comprehensive needs of older adults, especially those with multiple comorbidities. Fragmented healthcare systems may result in poor coordination between physical and mental health services, leading to delayed diagnosis, inadequate treatment, and suboptimal clinical outcomes.

In response to these challenges, integrated care models have emerged as a promising strategy to improve the management of depression and other chronic conditions. These models highlight coordinated, patient-centered care and commonly include components such as care coordination, shared decision-making, systematic symptom monitoring, patient education, and behavioral interventions [5]. The World Health Organization’s Integrated Care for Older People (ICOPE) framework is a multidomain geriatric care pathway rather than a stand-alone psychological treatment. It encompasses comprehensive assessment, individualized care planning, self-management support, and the use of multidisciplinary teams supported by integrated information systems and strong community linkages [4].

Despite increasing interest in integrated care, significant variability exists in how these models are designed and implemented across healthcare systems [6,7]. Several prior reviews on collaborative or integrated care for depression have shown that these models improve depression outcomes compared with usual care, but these reviews predominantly included general adult populations rather than focusing specifically on older adults [5]. More recently, Tops et al. reviewed integrated care models for older adults with depression and physical comorbidity, identifying 13 care models and emphasizing their components, implementation strategies, and clinical and organizational outcomes [8]. Liu et al. specifically evaluated ICOPE-based interventions and found that these interventions were associated with reduced depressive symptoms [9]. A recent meta-analysis also examined the components of collaborative care in adult primary care and identified structured therapeutic treatment, including manual psychotherapy and family involvement, as an important contributor to improved depression outcomes [10].

Various integrated care models may differ in their professional composition, care manager roles, use of psychological therapies, integration with chronic disease management, intensity and duration, delivery setting, and use of digital or telehealth support. Studies also vary in their target populations, comparator conditions, depression measures, follow-up periods, and definitions of clinical improvement. A structured scoping review that synthesizes these dimensions is therefore needed to make the evidence more clinically and conceptually interpretable.

Accordingly, this scoping review aimed to: (1) describe how integrated care was designed and operationalized; (2) characterize team composition, care manager and specialist roles, provider qualifications, intervention components, delivery modality, intensity, and follow-up; and (3) summarize the direction, durability, and magnitude of depression-related outcomes.

## 2. Methods

### 2.1. Review Design and Reporting Framework

This scoping review was guided by the Joanna Briggs Institute (JBI) methodology for scoping reviews and is reported in accordance with the Preferred Reporting Items for Systematic Reviews and Meta-Analyses extension for Scoping Reviews (PRISMA-ScR) [11,12]. The completed PRISMA-ScR checklist is provided in Appendix A. The protocol was not registered or published before the review was undertaken.

### 2.2. Inclusion and Exclusion Criteria

Eligibility criteria were organized according to the Population, Concept, Context framework [11,12]. The population was older adults aged 65 years or older or mixed adult populations that included older adults. The concept was an integrated or collaborative care intervention in which depression was a treatment target or reported outcome. Eligible contexts included primary care, community and aged-care services, home health care, chronic disease services, and technology-supported care. Eligible studies used an interventional study design (RCT, cluster RCT, or quasi-experimental study) and were published in the English language between 2006 and February 2026. The timeframe was selected to capture current models of integrated care. Studies were excluded if they: (1) did not report depression outcomes; (2) did not include older adults; (3) were observational studies, reviews, or qualitative studies; or (4) were study protocols without outcome data.

### 2.3. Information Sources and Search Strategy

A literature search was conducted in PubMed and supplemented by Google Scholar. PubMed was selected as the principal biomedical information source because it includes MEDLINE as its primary component, together with citations from additional life science journals. Google Scholar was used as a supplementary interdisciplinary search source to capture additional relevant studies not indexed in PubMed, given the interdisciplinary nature of the topic. Searches covered January 2006 to February 2026, and the last search update was completed in February 2026. Search results were exported to an Excel screening file. The following search terms were used: “integrated care” OR “collaborative care” OR “coordinated care” OR “multidisciplinary care” OR “behavioral health integration”) AND (“depression” OR “depressive disorder” OR “geriatric depression” OR “mental health”) AND (“older adults” OR “elderly” OR “aging”).

The terminology used to describe integrated approaches varies across the literature; therefore, the search terms were broad. However, these terms are not fully interchangeable. In this review, integrated care is used as an overarching term for organized approaches that bring together mental health care with primary, physical health, community, or social and behavioral services to provide more continuous and patient-centered management. Collaborative care is considered a specific integrated care model that typically involves a multiprofessional team, a designated care manager, systematic monitoring of symptoms and treatment, and consultation or supervision by a mental health specialist. Coordinated care refers to the processes used to facilitate communication, referrals, follow-up, information sharing, and continuity across providers or services. Multidisciplinary care describes the involvement of professionals from more than one discipline.

### 2.4. Selection Process

Duplicate and overlapping records were removed before formal screening. One reviewer (L.S., supervising author) performed the literature search and completed the initial title-and-abstract screening. Reports considered potentially eligible were retrieved and distributed among four reviewers (M.H., J.P., C.S., and M.D.). Each reviewer assessed the full-text reports assigned to them using the prespecified inclusion and exclusion criteria. Questions concerning eligibility were discussed with the supervising author and resolved by referring to these criteria. Full-text reports were not independently assessed by two reviewers. No automation tools were used during study selection.

### 2.5. Data Abstraction and Synthesis

Data from all eligible studies were extracted by the five reviewers using a structured data extraction template. Each study was extracted by the reviewer responsible for reviewing that report. In cases of questions, the supervising author (L.S.) resolved them through discussions. Data were extracted on author and year of publication, country or setting, population, design and sample size, intervention components, follow-up duration and findings on depression-related outcomes (Supplementary Table S1). Depression outcomes were the primary outcomes of interest. When reported, additional outcomes directly relevant to the integrated care model were also charted, including physical-health indicators, medication adherence, self-management, functional status, quality of life, and social outcomes. These additional outcomes were considered secondary outcomes and were not required for study eligibility. Data extraction was not performed independently in duplicate, and reviewer agreement was therefore not assessed. The extracted data was synthesized and presented in narrative summaries.

### 2.6. Risk of Bias and Certainty Assessment

No formal assessment on risk of bias or certainty of evidence was performed because this scoping review was intended to assess the design and key features of integrated care interventions rather than determine comparative effectiveness. Consequently, the synthesis is not quality weighted and should not be interpreted as a formal assessment of comparative effectiveness or certainty of evidence.

### 2.7. Synthesis Methods

A structured descriptive synthesis was developed. Sources of evidence were compared across four domains: (1) definition and organizational structure of integrated care; (2) workforce, qualifications, case manager and specialist roles, as well as intervention content; (3) delivery intensity, monitoring, technology, and implementation supports; and (4) evaluation design, outcome measure, assessment timing, magnitude of difference, and assessment of moderators and mediators.

Comparator conditions may include usual care, enhanced usual care, active specialty referral, or another service model. The specific services reported within each comparator were recorded because usual care varied across healthcare settings and studies.

## 3. Results

**Figure 1:**
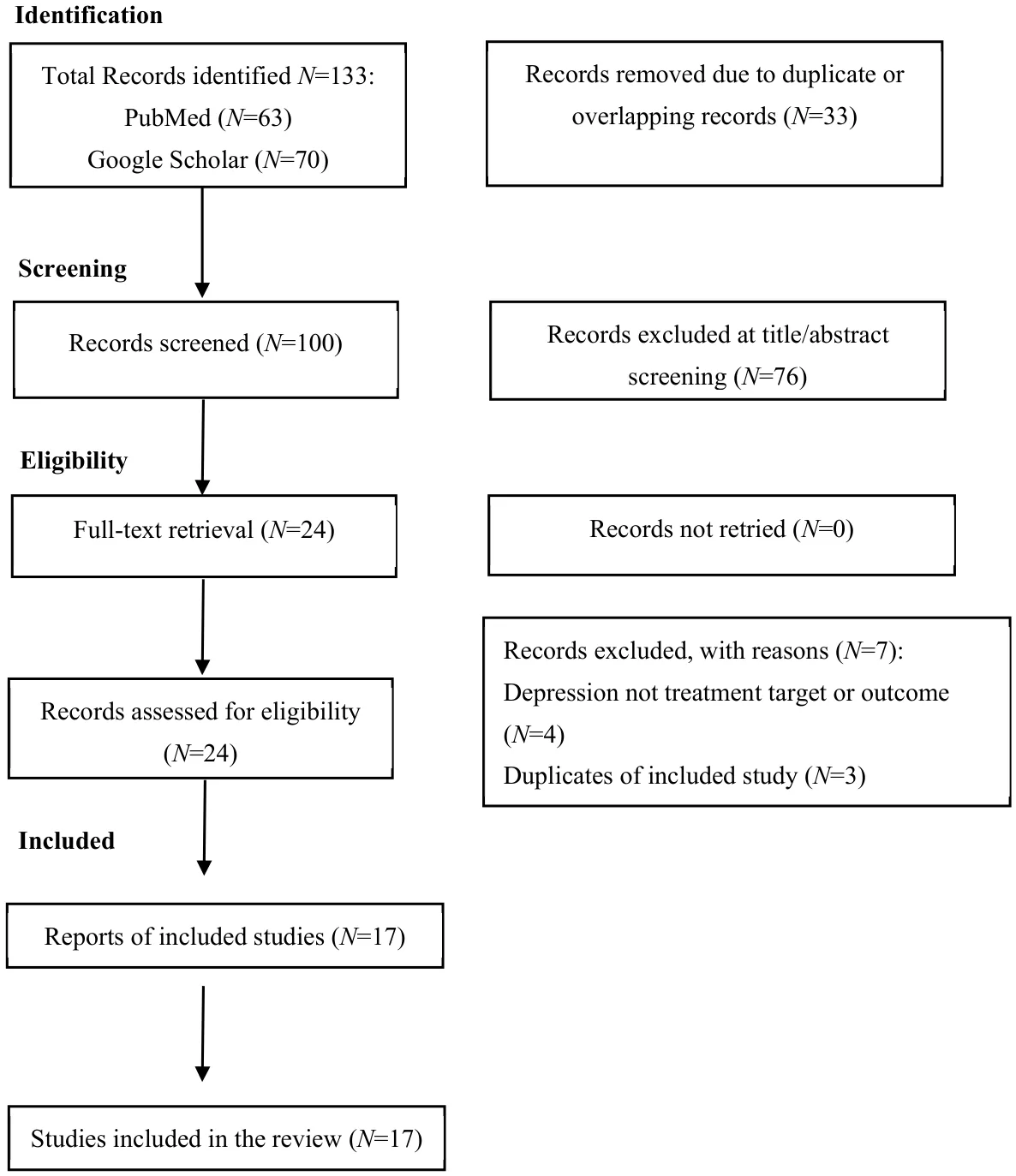
PRISMA-ScR flow diagram of study selection.

The database search yielded 133 records in total (PubMed = 63; Google Scholar = 70). After removal of 33 duplicate or overlapping records, 100 records underwent title and abstract review. A total of 76 studies were excluded, leaving 24 for full-text review. Following full-text review, seven studies were excluded because depression was not a treatment target or reported outcome (*n* = 4) or because the report duplicated an already included study (*n* = 3). Seventeen studies were included in the final synthesis (Figure 1).

### 3.1. Study Characteristics

The 17 included studies were conducted in multiple countries, including Australia, Brazil, China, the Netherlands, Norway, Singapore, Taiwan, the United Kingdom, and the United States, and spanned a range of healthcare settings, such as primary care clinics, community health centers, community and aged-care programs, multisector service organizations, and home healthcare services. Participants commonly had depression alongside chronic medical conditions including diabetes, hypertension, heart failure, or chronic obstructive pulmonary disease. Sample sizes ranged from 34 to 3416 participants. Of the 17 studies, 12 primarily targeted geriatric populations, although the operational definition of older age varied across studies, with some using a minimum age of 60 years and others using 65 years or older adults. The remaining five studies enrolled populations with broader age ranges [13,14,15,16,17]. These mixed-age studies were included because their samples contained older adults and they evaluated integrated care relevant to the review question.

### 3.2. Operationalization of Integrated Care

Integrated care was operationalized as a family of overlapping service models rather than a single intervention. Across 17 studies, several consistent components of integrated care models were identified, including multidisciplinary care teams; structured psychological interventions; integration with chronic disease management, community, aged-care, or multisector models combining health and social services; and, in some cases, home-based or technology-supported care. Table 1 identifies the key components and the mechanism used to connect providers or services for each study. To facilitate comparison across heterogeneous interventions, the included studies were organized according to the integrated care model types, including primary-care collaborative or behavioral depression care, depression care integrated with chronic-disease management; primary care with direct specialty integration or consultation; and community, aged care, or home-based care. These categories are not mutually exclusive, as several studies incorporated characteristics of more than one category.

#### 3.2.1. Multidisciplinary Teams

Multidisciplinary, team-based care was a significant feature of nearly all included studies (*n* = 15/17). A care manager, case manager, coordinator, integrated care manager, or functionally equivalent coordinating worker was described in 15 of 17 studies. However, these roles were not professionally uniform. They included registered or mental health nurses, psychological wellbeing practitioners, research coordinators, village lay workers, bilingual promotoras or community health workers, social workers, and primary care case managers.

Primary care physicians or general practitioners had an explicit role in treatment, prescribing, consultation, or liaison in all studies. Four studies explicitly identified nurses as direct intervention providers or coordinators. Psychology providers, psycho-therapists, or psychiatrists were reported in seven studies, and licensed co-located mental health or substance-use providers were reported in one. Six studies used community health workers, lay workers, social workers, peer supporters, student health communicators, or community-sector staff. Specialist involvement ranged from direct treatment to remote consultation or referral. PRISM-E placed licensed mental health providers inside primary care clinics and compared this with enhanced referral to physically separate specialty clinics [23]. COACH used remote psychiatric consultation by telephone to support village physicians and lay workers [22]. The IMPACT trial brought a geriatric psychiatrist into two joint consultations with the patient and general practitioner [24]. Other programs used specialist supervision of non-specialist providers, stepped referral for severe symptoms, or ad hoc consultation (Table 1).

#### 3.2.2. Intervention Components, Provider, and Dose

Five studies explicitly included behavioral activation in depression treatment. Three studies reported problem-solving treatment, and one study included cognitive behavioral therapy. Psychoeducation, adherence counseling, or structured depression self-management was reported in five studies, and antidepressant initiation, adjustment, or protocol-based medication management was reported in six. Additional programs described psychosocial support without sufficient details. Table 2 summarizes each treatment, the provider who delivered it, and the planned number and mode of contacts.

Treatment intensity varied substantially. Examples included three in-person and two telephone contacts over 12 weeks [13], approximately six case-manager sessions over 7–8 weeks in CASPER Plus [18], six weekly promotora sessions followed by three monthly boosters [15], eight weekly 35 min telephone problem-solving sessions plus daily telemonitoring in I-TEAM [25], eight weekly 30 min behavioral-activation sessions [19], 16 tailored contacts over 12 months in COMRADE [30], a 17-week home program in PROACTIVE [26], and up to 18 contacts over 12 months in CIC-PDD [17]. For several models, especially community-based programs, the patient-level dose and between-session contact were not fully reported.

Fourteen studies combined depression care with physical health, chronic disease management, medication adherence, activity, or lifestyle management. Seven included social, functional, welfare, peer, family, or community resource components, and one explicitly included spiritual wellbeing [27]. These components are theoretically relevant because depression can impair health behavior and medical control, while disability, symptoms, loneliness, and poor health can sustain depression. The studies rarely separated the direct effect of psychotherapy from effects mediated through better physical health, activity, self-management, or social connection. Only one study reported that reduced loneliness and stepped care might serve as mediator [26].

According to the WHO ICOPE, the assessment is the entry point, while the linked care actions constitute the intervention. In a trial implementing the WHO ICOPE pathway, screening for intrinsic-capacity decline was followed by comprehensive assessment, an individualized care plan, referral or treatment for identified needs, and proactive follow-up [28].

Technology was a common feature in integrated care models. Nine studies used telephone, telemonitoring, tablet, WeChat, or other digital communication as a primary or adjunct delivery mechanism. These tools supported remote psychotherapy, symptom and physiologic monitoring, adherence checks, psychiatric consultation, booster contacts, case communication, or follow-up.

Training and supervision were heterogeneous. Some interventions were delivered by licensed professionals within their usual scope; others used task-sharing after study-specific training. Reported examples included two-day collaborative-care training for psychological wellbeing practitioners, structured training for village lay workers in depression–hypertension self-management and psychosocial care, training and supervision of promotoras and community health workers, and specialist-supported case conferences. Many reports did not provide enough information about competency assessment, fidelity monitoring, or ongoing supervision to determine the resources required to reproduce the program.

#### 3.2.3. Outcome Measures and Assessment Timing

Table 3 presents the outcome measures and effect estimates of each study. Nine studies used individual participant randomization; six used cluster randomization at the practice, clinic, village, or program level; and two used a quasi-experimental design. The designs therefore differed not only in sample size but also in the level at which the intervention was assigned and analyzed.

Patient-reported symptom scales included the PHQ-9, Geriatric Depression Scale, Beck Depression Inventory, Center for Epidemiologic Studies Depression Scale, and Brief Symptom Rating Scale. Three studies also used clinician-rated Hamilton depression measures. Some studies used mental health-related quality of life or functional limitation.

Assessment schedules ranged from end-of-treatment measurements at approximately 12 weeks to follow-up at 18 months. Twelve studies evaluated whether benefit persisted at or beyond 12 months.

### 3.3. Direction, Durability, and Magnitude of Findings

Most studies (n = 13/17) reported improvements in depressive symptoms among participants receiving integrated care interventions compared with usual care. Among them, 11 studies reported a favorable result for integrated care, and two showed a favorable early result that was not maintained [16,18]. Among the remaining four studies, two found no significant between-group difference [15,24], one favored the enhanced specialty-referral comparator for symptom severity [23], and one had mixed findings according to which a short-term functional benefit was not sustained [19].

The magnitude of benefit varied. For example, the COACH trial demonstrated significant reductions in depression scores among older adults with hypertension receiving collaborative care in rural China [22]. Similarly, the COMRADE trial showed that intervention group had significantly greater improvement in depressive symptoms measured by the PHQ-9 (−3.39 vs. −0.90; *p* = 0.01) than usual care at 12-month follow-up [14]. In the I-TEAM intervention, participants receiving the integrated telehealth intervention demonstrated significantly greater reductions in depression compared to UC+P at both 3 months (PHQ-9: 7.4 vs. 13.6, *p* = 0.01; HAM-D: 9.8 vs. 18.6, *p* = 0.02) and 6 months (PHQ-9: 7.9 vs. 14.1, *p* = 0.05) [25]. The PROACTIVE trial in Brazil found significantly higher rates of recovery from depression (PHQ-9 score < 10) at the 8-month and 12-month follow-up assessments, comparing the collaborative care psychosocial intervention versus enhanced usual care [26,31].

Benefits were not uniform. The CollAborative care for Screen-Positive EldeRs (CASPER) plus trial in the UK showed a significant effect in favor of collaborative care, with an effect size of 0.34 at 4 months. However, the treatment effect was not maintained in the longer term at 12 months or 18 months [18]. Ell et al. and Kvalbein-Olsen et al. found improvement over time without a significant between-group difference [15,24]. In the PRISM-E study, enhanced specialty referral care produced significantly greater reductions in depression severity compared with integrated care (mean difference = 2.8, 95% CI: 1.0–4.5; *p* = 0.003) among participants with major depression, although remission and functional outcomes were broadly comparable [23]. These findings demonstrate that comparator intensity and the services already present in usual care can materially affect the observed contrast.

Several studies examined subgroups, dose response, or effect modification. For example, CASPER Plus examined the receipt of six or more sessions and did not find any statistically significant difference in benefit [18]. COMRADE reported no clear differences in glycemic response by race or gender [14], and CIC-PDD reported greater improvement in self-health management among rural participants [17].

### 3.4. Risk of Bias and Certainty of Evidence

The risk of bias and certainty of evidence were not formally assessed in this review. Consequently, results should be interpreted as scoping evidence of published intervention findings focusing on the design and key features rather than as a formal estimate of comparative effectiveness.

## 4. Discussion

This scoping review identified substantial variation in how integrated care for depression management among older adults was defined, staffed, delivered, and evaluated. Outcome evaluation was heterogeneous. Eleven studies favored integrated care, and two studies found benefits limited to earlier time points, while four studies found no advantage or favored a strong specialty comparator.

Prior reviews have reached mixed conclusions. In a broad review of collaborative care for depression and anxiety across adult populations, Archer et al. reported overall benefits compared with usual care, although effects varied across studies and outcomes [5]. In contrast, Frost et al. focused on older adults with depression or anxiety and physical or cognitive comorbidities affecting functioning and found little evidence that collaborative care improved depressive symptoms, functioning, or quality of life [32]. Liu et al. reported a pooled improvement in depressive symptoms for ICOPE-based interventions but rated the cumulative evidence as very low certainty and reported substantial heterogeneity [9].

The present review used a broad operationalization of integrated care, including primary care, collaborative care, chronic disease integration, community and social service models, home telehealth, and multidomain geriatric pathways. Multidisciplinary teamwork and care coordination emerged as main components of integrated care. Care managers, nurses, primary care providers, and mental health specialists worked collaboratively to deliver patient-centered care, supporting adherence and continuity across settings. Although a care manager function was common, it was fulfilled by different personnel. In addition, specialist support ranged from co-located licensed treatment to telephone consultation, supervision, case conferences, or referral. These models therefore cannot be translated into a single staffing formula.

The component analysis also supports structured behavioral treatments. Many interventions simultaneously addressed medication adherence, chronic disease management, physiologic monitoring, mobility, activity, social isolation, welfare needs, or functional capacity. These elements may reduce depression indirectly by improving health, autonomy, and social connection, while improvement in depression may, in turn, support adherence and self-management.

Several limitations should be considered. First, there was substantial variability in intervention design, duration, and outcome measures, limiting direct comparison across studies. Five of the 17 included studies enrolled broad or mixed-age adult populations rather than populations composed primarily of older adults, and age-stratified outcome data were generally unavailable. When interpreting the results from the mixed-age studies, caution should be exercised in terms of how integrated care is attributed specifically to older adults. The follow-up periods of the studies were relatively short, with 3 to 18 months of follow-up, limiting conclusions about long-term effectiveness.

In addition, this review was limited to two information sources and English-language publications, which may have excluded relevant studies. Although PubMed includes MEDLINE and Google Scholar broadened retrieval, other databases, such as PsycINFO, Embase, CINAHL, and CENTRAL, were not searched. Therefore, the evidence base may not be complete. Furthermore, study selection was not conducted using independent duplicate screening. The literature search and initial title-and-abstract screening were conducted by one reviewer (supervising author), after which potentially eligible full-text reports were divided among five reviewers, with each report assessed by one assigned reviewer. Data extraction was also not performed independently in duplicate. These procedures may have increased the risk of selection or extraction errors and reduced the reproducibility of the review. Lastly, no formal quality assessment was performed on the risk of bias or certainty of evidence.Despite these limitations, the review has practical implications. From a clinical perspective, these findings support the integration of mental health services into primary care for older adults, focusing on those with comorbid conditions. Care coordination, behavioral therapies, and multidisciplinary collaboration appear to be strong components of effective models. From a systems perspective, implementation requires investment in workforce development, including care managers and nursing roles, as well as infrastructure to support coordination and communication. For researchers, future work should focus on implementation strategies, sustainability, cost, equity, and long-term outcomes, especially in real-world health systems serving older adults with comorbidity.

## 5. Conclusions

This scoping review revealed integrated care for depression management in older adults as primary care-collaborative, chronic disease management-integrated, and multidisciplinary geriatric care models. There is substantial heterogeneity in how these models are organized, staffed, delivered, and evaluated. The majority of the studies reported favorable depression outcomes from integrated care models. Future research should focus on identifying the effective components of integrated care models, staff qualifications and care delivery, and moderator and mechanism analyses. Such information would make future evidence more useful to clinicians, administrators, and health-system planners. As the population continues to age, expanding integrated care approaches will be essential to effectively addressing the growing burden of late-life depression.

## Figures and Tables

**Table 1:** Organizational structure, staffing, and specialist arrangements in the included studies by integrated care model type.

Study	Model/Setting	Primary Care Staff	Care Manager/Coordinator	Mental Health Specialist	Other Personnel	Integration and Training
**A. Primary-care collaborative or behavioral depression-care models**						
Bosanquet et al. (2017) [18]	Collaborative care (including BA); UK primary care	GPs retained medical care	NHS band 5 psychological wellbeing practitioners; trial-specific training	Specialist supervision/medication management	NR	Case manager delivered BA and liaised with GP by face-to-face or telephone contact
Janssen et al. (2023) [19]	BA; primary care	GP/primary care centers	Trained mental health nurses	Mental health nurse was the direct BA provider	NR	Eight-session protocol
Almeida et al., (2021) [20]	Telephone-supported BA; rural community	Usual primary care/GP	No separate coordinator named	Trained psychologist; remote by telephone	NR	Psychologist-supported self-managed BA alongside usual care
Ng et al., (2020) [21]	GP-led collaborative care; primary care	Trained GPs	Case management coordinator	Referral or consultation with mental health specialists	NR	Protocol-based antidepressant treatment, referral, and case management over six months
**B. Depression care integrated with chronic-disease management**						
Bogner et al. (2012) [13]	Integrated depression–diabetes management; primary care	Primary-care physicians	Two research coordinators: one master’s-level and one bachelor’s-level; trained in diabetes pharmacotherapy	NR	NR	Coordinators provided education and adherence/clinical monitoring and communicated with physicians; in-person plus phone
Cummings et al. (2019) [14]	Tailored diabetes–depression/distress care; primary care	Primary-care team/routine medical care	Nurse care manager for lower severity lifestyle coaching	Psychologist or doctoral clinical-psychology trainee for CBT/PST	Community health worker support	Severity-stratified assignment; diabetes guideline integration; face-to-face and telephone contacts
Wang, Y., et al. (2025) [17]	Integrated diabetes–depression care; community health centers	PCPs and diabetes specialist	Primary care case managers	Psychiatrist/psychotherapist	Student health communicators	Structured manual, interprofessional case conferences, measurement-based care, and WeChat follow-up
Chen et al. (2022) [22]	Depression–hypertension collaborative care; rural primary care	Village primary-care physicians	Village lay workers	Psychiatrists provided remote telephone consultation	Family involvement	Village lay workers trained in self-management, psychosocial assessment, care planning, psychoeducation, confidentiality, and ethics
**C. Primary care with direct specialty integration or consultation**						
Krahn et al. (2006) [23]	Co-located integrated care vs. external specialty referral; primary care	Primary-care physicians	Licensed mental health/substance-use provider delivered and coordinated care	Licensed providers co-located in clinic	NR	In-clinic integration with enhanced referral
Kvalbein-Olsen et al. (2025) [24]	Joint primary care specialty consultation	GPs	No separate care manager	Geriatric psychiatrist attended two joint consultations	Municipal and social services as needed	Shared individualized plan followed by GP-led consultations; specialist access was direct but time-limited
**D. Community, aged-care, or home-based integrated care models**						
Ell et al. (2017) [15]	Promotora-assisted integrated care; safety-net primary care	Patient-centered primary-care team	Three trained bilingual promotoras (lay community health workers)	NR	Community resource links	Promotoras were trained and supervised to deliver psychoeducation/problem solving, chronic-disease self-management, and patient activation
Izquierdo et al. (2018) [16]	Community coalition implementation; multisector programs	Health-sector programs within coalition	Coalition/program leads; individual care manager role varied	Mental health staff available across participating programs	Community, social service, faith, and other sector staff	Coalition planning and technical support were used to implement collaborative care; personnel were heterogeneous
Gellis et al. (2014) [25]	Home-health telehealth; Hospital-affiliated home healthcare setting	Home-health services and PCP; PCP prescribed antidepressants	Telehealth nurse	NR	NR	Nurse delivered daily monitoring and PST and communicated with PCP.
Seward et al., (2025) [26]	Task-shared stepped collaborative care; primary care and home	Primary care clinics	Community health workers	Specialist training and supervision	Community health workers	Non-mental health workers delivered home sessions with tablet support within a stepped-care model.
Liao et al. (2022) [27]	Nurse-led multidomain geriatric care; community	Medical services accessed as required	Trained registered nurses delivered the integrated care model	NR	Social welfare and safety services	Nurse assessment linked to health, function, spiritual wellbeing, welfare, and elder-abuse prevention actions
Wang, N., et al. (2024) [28]	WHO ICOPE; community primary care	Community primary-care team	Integrated care managers	Referral based on assessed psychological needs	Health and social service links	Screening was linked to comprehensive assessment, individualized planning, referral, and proactive follow-up
Liu et al. (2026) [29]	Stepped aged-care/mental-health collaboration; community	Aged-care and mental health service units	Trained social workers	Specialist mental health referral for severe cases	Trained peer supporters	Social workers delivered psychosocial care; peers supported maintenance and relapse prevention

Abbreviations: BA = behavioral activation; CBT = cognitive behavioral therapy; GP = general practitioner; ICOPE = Integrated Care for Older People; NR = not reported; PCP = primary care provider; PST = problem-solving treatment. Note: Interventions were grouped according to their key organizational structure. Categories are descriptive and are not mutually exclusive. Several studies incorporated characteristics of more than one category.

**Table 2:** Intervention content, provider and delivery.

Study	Depression-Specific Treatment	Provider	Planned Dose/Contact	Physical Health or Lifestyle Content	Social and Functional Support	Monitoring, Technology, or Supervision
Bogner et al. (2012) [13]	Education, adherence support, and antidepressant treatment coordination	Research coordinators with physicians	3 × 30 min in-person sessions plus 2 × 15 min telephone contacts over 12 weeks	Diabetes medication adherence and clinical monitoring	NR	Medication/adherence monitoring and physician communication
Cummings et al. (2019) [14]	CBT/PST for higher symptoms; lifestyle counseling for lower symptoms	Psychologist/doctoral trainee or nurse care manager	16 sessions over 12 months; in-person and telephone	Diabetes self-care, diet/activity and guideline-based medical care	Community health worker support	Severity-tailored allocation and ongoing monitoring
Ell et al. (2017) [15]	Psychoeducation and problem-solving strategies	Trained promotoras	6 weekly sessions plus 3 monthly telephone boosters	Chronic disease self-management	Patient activation, provider communication, and community resources	In-person or telephone sessions with booster contact and supervision
Izquierdo et al. (2018) [16]	Collaborative depression care; patient-level therapy content varied	Health and community program staff	Coalition-level implementation; no details on patient-level dose	Varied by participating program	Community engagement and multisector resources	Community engagement/planning vs. individual technical assistance
Wang, Y., et al. (2025) [17]	BA and measurement-based depression care; specialist treatment as indicated	Case managers and psychiatrist/psychotherapist	Up to 18 sessions over 12 months	Diabetes self-management and clinical monitoring	Health communication support	WeChat follow-up, symptom/HbA1c monitoring, case conferences, and stepped treatment
Krahn et al. (2006) [23]	Assessment, counseling, case management, psychotherapy, and pharmacological treatment	Licensed co-located mental health/substance-use provider	NR	Coordination with primary medical care	NR	Communication about the evaluation and treatment plan between clinicians and the primary care providers; appointment with mental health or substance abuse provider after the primary care provider visit
Chen et al. (2022) [22]	Psychoeducation and algorithm-guided depression management	Village physician and lay worker with psychiatrist consultation	Contacts over 12 months	Hypertension self-management, adherence, and control	Family and contextual support	Structured symptom/clinical monitoring; remote psychiatric consultation
Kvalbein-Olsen et al. (2025) [24]	Individualized depression plan; medication adjustment possible	GP and geriatric psychiatrist	2 joint consultations followed by GP-led consultations	Medical review and service coordination as needed	Municipal/social services as needed	Shared care plan
Bosanquet et al. (2017) [18]	BA plus collaborative medication management/GP liaison	Psychological wellbeing practitioner case manager	Average of 6 sessions over 7–8 weeks; up to 8–10 offered	NR	NR	Face-to-face or telephone follow-up; specialist-supervision framework
Gellis et al. (2014) [25]	Telephone PST plus antidepressant management	Telehealth nurse and PCP	8 weekly 35 min PST sessions over 3 months plus daily monitoring	Daily physiological, symptom, weight, and medication monitoring	NR	Home telemonitoring and nurse–PCP communication
Janssen et al. (2023) [19]	BA	Trained mental health nurses	8 weekly 30 min sessions	Activity engagement and functioning	NR	NR
Seward et al., (2025) [26]	Psychoeducation and BA	Community health workers with specialist supervision	17-week home program	Activity scheduling	Social activation and loneliness-related mechanisms	Tablet-supported task sharing and stepped care
Liao et al. (2022) [27]	Mental-wellbeing support, such as dementia and delirium management	Trained nurses	12-week intervention; outcomes through 18 weeks	Health-problem management and activities of daily living	Spiritual wellbeing, social welfare services, and elder abuse prevention	Multidomain assessment linked to individualized actions and referrals
Wang, N., et al. (2024) [28]	No stand-alone psychotherapy; psychological needs addressed within ICOPE plan	Integrated care manager/team	6-month program	Mobility, nutrition, cognition, sensory and other health needs	Social care and caregiver support	ICOPE screening, comprehensive assessment, individualized plan, referral and follow-up; online/offline support
Almeida et al., (2021) [20]	Self-managed BA	Trained psychologist	Telephone support over 8 weeks	Activity engagement; no disease-specific management reported	NR	Telephone delivery
Ng et al., (2020) [21]	Antidepressant treatment, referral and case management	Trained GPs	6-month collaborative-care period	Routine medical care	NR	Protocol follow-up and specialist referral
Liu et al. (2026) [29]	Evidence-based psychosocial strategies with stepped referral	Trained social workers; peers for maintenance	Intensity varied by symptom severity; follow-up of 2–12 months	NR	Peer support, loneliness/social support, and aged-care services	Stepped care; specialist referral for severe cases

Abbreviations: BA = behavioral activation; NR = not reported.

**Table 3:** Evaluation design, outcome assessment and magnitude.

Study	Design	Depression Measure	Assessment Timing	Direction and Durability	Key Magnitude	Moderators or Mediators
Bogner et al. (2012) [13]	Individual RCT	PHQ-9	6 and 12 weeks	Favorable at 12 weeks	Remission (PHQ-9 <5): 58.7% vs. 30.7% at 12 weeks	NR
Cummings et al. (2019) [14]	Individual RCT	PHQ-9	12 months	Favorable at 12 months	PHQ-9 change of −3.39 vs. −0.90, *p* = 0.01	No clear race or gender differences in glycemic response
Ell et al. (2017) [15]	Individual RCT	PHQ-9	6 and 12 months	No significant between-group difference	Both groups improved; between-group difference not significant	NR
Izquierdo et al. (2018) [16]	Cluster RCT; community programs	PHQ-8, mental health-related quality of life, and mental wellness	6 and 12 months	Favorable at 6 months, but early benefit not maintained at 12 months	At 6 months: 9.2% lower poor mental health quality of life and 12.8% more mental wellness improvement; no significant 12-month difference	Older-adult subgroup; patient-level intervention exposure varied across programs
Wang, Y., et al. (2025) [17]	Cluster RCT; community health centers	SCL-20; SF-12 mental component	6 and 12 months	Favorable at longest follow-up	≥50% SCL-20 reduction: 62.1% vs. 31.0%; risk difference of 31.03% (95% CI 21.85–40.21)	Rural participants showed greater improvement in self-health management
Krahn et al. (2006) [23]	Individual RCT	CES-D, MCS of SF-36, and MINI	3 and 6 months	Comparator favored for symptom severity	Enhanced specialty referral mean symptom difference of 2.8 (95% CI of 1.0–4.5), *p* = 0.003, for major depression; remission and functional change were comparable	Combination of talk therapy and pharmacotherapy worked better in the enhanced-specialty referral model than in the integrated care model among patients with major depression
Chen et al. (2022) [22]	Cluster RCT; villages	HDRS clinician-rated; PHQ-9 screening	12 months	Favorable at 12 months	Standardized between-group HDRS difference of −1.43 (95% CI −1.71 to −1.15), *p* < 0.001	NR
Kvalbein-Olsen et al. (2025) [24]	Cluster RCT; GP practices	PHQ-9	6, 12, and 18 months	Both groups showed significant mean PHQ-9 score reductions at 18 months but no significant between-group difference	PHQ-9 reductions of 3.4 vs. 4.0 at 18 months; group differences not significant	NR
Bosanquet et al. (2017) [18]	Individual pragmatic RCT	PHQ-9 and depression severity	4, 12, and 18 months	Favorable at 4 months but early benefit not maintained at 12 and 18 months	Mean difference of 1.92 points at 4 months; not significant at 12 or 18 months	Receipt of ≥6 sessions was explored; larger effect was not statistically definitive
Gellis et al. (2014) [25]	Individual RCT	PHQ-9 and HAM-D clinician-rated	3 and 6 months	Favorable at 6 months	At 6 months: PHQ-9 7.9 vs. 14.1, *p* = 0.05; HAM-D 10.4 vs. 17.4, *p* = 0.05	NR
Janssen et al. (2023) [19]	Cluster RCT; primary care centers	PHQ-9; WHODAS functioning	End of treatment and 12 months	Favorable at the end of treatment, but benefit was not sustained	WHODAS difference of −3.62 at end of treatment, *p* = 0.01; not significant at 12 months	A significant moderating effect of MoCA on functioning, indicating that the differences were driven by those with no cognitive impairment
Seward et al., (2025) [26]	Cluster RCT; primary care clinics	PHQ-9; recovery defined as <10	8 and 12 months	Favorable at 8 months	Recovery of 62.5% vs. 44.0% at 8 months; OR of 2.33 at 12 months, *p* < 0.001	Reduced loneliness and stepped care might serve as a mediator
Liao et al. (2022) [27]	Quasi-randomized trial	CES-D, BSRS-5 and related outcomes	12 and 18 weeks	Favorable at 18 weeks	Interaction effect: CES-D −4.76 (95% CI −7.39 to −2.14), *p* < 0.001 at 18 weeks	NR
Wang, N., et al. (2024) [28]	Individual RCT	GDS-5	6 months	Favorable at 6 months	Adjusted difference in GDS-5 of 0.09 (95% CI 0.03–0.14), *p* < 0.05	NR
Almeida et al., (2021) [20]	Individual RCT	PHQ-9 and GAD-7	26 and 52 weeks	Favorable at 52 weeks	PHQ-9 mean difference of 1.0 at 52 weeks, *p* = 0.048	NR
Ng et al., (2020) [21]	Individual RCT	HDRS-17 clinician-rated; GDS and BDI self-report	3, 6, and 12 months	Favorable at 3, 6, and 12 months	1.5-point greater HDRS-17 change at 6 months; benefit also observed at 3 and 12 months	NR
Liu et al. (2026) [29]	Pragmatic quasi-experimental allocation by service area	PHQ-9; anxiety and loneliness scales	2–12 months	Favorable at 12 months	Adjusted PHQ-9 mean difference of −1.65, *p* < 0.001	Treatment effect was stronger among people with moderate to moderately severe symptoms and those with risk factors

Abbreviations: BDI = Beck Depression Inventory; BSRS = Brief Symptom Rating Scale; CES-D = Center for Epidemiologic Studies Depression Scale; GAD-7 = Generalized. Anxiety Disorder-7; GDS = Geriatric Depression Scale; HAM-D = Hamilton depression rating scales; NR = not reported; PHQ-9 = Patient Health Questionnaire-9; RCT = randomized controlled trial; SCL-20 = Symptom Checklist-20; WHODAS = World Health Organization Disability Assessment Schedule.

## Data Availability

This study is based on previously published literature, all of which is cited within the manuscript. No new data were generated or analyzed.

## Supplementary Materials

The following supporting information can be downloaded at: https://www.mdpi.com/article/10.3390/geriatrics11050126/s1, Table S1: Detailed study characteristics.

## Abbreviations

BSRS: Brief Symptom Rating Scale; CBT: Cognitive Behavioral Training; COACH: Chinese Older Adult Collaborations in Health; DDS: Diabetes Distress Scale; ED: emergency department; HAM-D: Hamilton Depression Rating Scale; ICM: integrated care model; I-TEAM: Integrated Telehealth Education and Activation of Mood; MCS: Mental Component Subscales; MINI: Mini-International Neuropsychiatric Interview; MMSE: Mini-Mental State Examination; PHQ: Patient Health Questionnaire; PST: problem-solving treatment; RCT: randomized controlled trial; SF: short-form survey; T2D: type 2 diabetes; UC+P: usual in-home care plus psychoeducation; WHODAS: World Health Organization Disability Assessment Schedule.

## Appendix A

**Table A1:** PRISMA-ScR Checklist.

Section	Item	PRISMA-ScR Checklist Item	Reported on Page Number
**TITLE**			
Title	1	Identify the report as a scoping review.	Title page
**ABSTRACT**			
Structured summary	2	Provide a structured summary that includes (as applicable): background, objectives, eligibility criteria, sources of evidence, charting methods, results, and conclusions related to the review questions and objectives.	Abstract
**INTRODUCTION**			
Rationale	3	Describe the rationale for the review in the context of what is already known. Explain why the review questions or objectives lend themselves to a scoping-review approach.	Introduction
Objectives	4	Provide an explicit statement of the questions and objectives being addressed with reference to their key elements (e.g., population or participants, concepts, and context) or other relevant key elements used to conceptualize the review questions and/or objectives.	Introduction, Methods. final paragraph
**METHODS**			
Protocol and registration	5	Indicate whether a review protocol exists; state if and where it can be accessed (e.g., a Web address); and if available, provide registration information, including the registration number.	Methods
Eligibility criteria	6	Specify characteristics of the sources of evidence used as eligibility criteria (e.g., years considered, language, and publication status), and provide a rationale.	Methods—Eligibility criteria
Information sources	7	Describe all information sources in the search (e.g., databases with dates of coverage and contact with authors to identify additional sources), as well as the date the most recent search was executed.	Methods—Information sources and search strategy
Search	8	Present the full electronic search strategies for at least one database, including any limits used, so that it could be repeated.	Methods—Information sources and search strategy
Selection of sources of evidence	9	State the process for selecting sources of evidence (i.e., screening and eligibility) included in the scoping review.	Methods—Selection process
Data charting process	10	Describe the methods of charting data from the included sources of evidence (e.g., calibrated forms or forms that have been tested by the team before their use, and whether data charting was done independently or in duplicate) and any processes for obtaining and confirming data from investigators.	Methods—Data collection process and data items
Data items	11	List and define all variables for which data were sought and any assumptions or simplifications made.	Methods—Data collection process and data items
Critical appraisal of individual sources of evidence	12	If done, provide a rationale for conducting a critical appraisal of included sources of evidence; describe the methods used and how this information was used in any data synthesis (if appropriate).	Not undertaken
Synthesis of results	13	Describe the methods of handling and summarizing the data that were charted.	Methods—Synthesis methods
**RESULTS**			
Selection of sources of evidence	14	Give numbers of sources screened, assessed for eligibility, and included in the review, with reasons for exclusions at each stage, ideally using a flow diagram.	Results—Study selection; Figure 1
Characteristics of sources of evidence	15	For each source of evidence, present characteristics for which data were charted and provide citations.	Results—Study characteristics; Table 1, Table 2 and Table 3, Supplementary Table S1
Critical appraisal within sources of evidence	16	If done, present data on critical appraisal of included sources of evidence.	Not applicable; critical appraisal was not undertaken
Results of individual sources of evidence	17	For each included source of evidence, present the relevant data that were charted that relate to the review questions and objectives.	Table 1, Table 2 and Table 3; Supplementary Table S1
Synthesis of results	18	Summarize and/or present the charting results as they relate to the review questions and objectives.	Results
**DISCUSSION**			
Summary of evidence	19	Summarize the main results (including an overview of concepts, themes, and types of evidence available), link to the review questions and objectives, and consider the relevance to key groups.	Discussion—paragraphs 1–4
Limitations	20	Discuss the limitations of the scoping review process.	Discussion—limitations paragraphs
Conclusions	21	Provide a general interpretation of the results with respect to the review questions and objectives, as well as potential implications and/or next steps.	Conclusion
**FUNDING**			
Funding	22	Describe sources of funding for the included sources of evidence, as well as sources of funding for the scoping review. Describe the role of the funders of the scoping review.	Funding

PRISMA-ScR = Preferred Reporting Items for Systematic Reviews and Meta-Analyses extension for Scoping Reviews [12].
