# Supplementary material for: A Scoping Review of Integrated Care Models for Managing Depression in Older Adults
Source: Geriatrics (Basel). 2026 Sep 8;11(5):126. doi: 10.3390/geriatrics11050126 (PMC13600076; doi:10.3390/geriatrics11050126)
Supplement: Supplementary file 1 [file geriatrics-11-00126-s001.zip › geriatrics-4278281-supplementary.pdf]

Supplementary Table S1. Detailed study characteristics

Study (year)	Setting/ Country	Population	Design / sample size	Intervention (integrated-care model components) and comparator	Follow-up	Main depression-related findings
Bogner et al. (2012) [13]	Primary care practices in Philadelphia, USA	Adults ≥30 years with T2D and depression who were prescribed an oral hypoglycemic medication and antidepressant	RCT; *N*=180 patients (integrated care intervention vs usual care)	Intervention: integrated care management model:<br>Care manager collaborated with physicians<br>Education about diabetes and depression treatment<br>Monitoring medication adherence and clinical status<br>In-person and telephone sessions to support adherence to antidepressant and diabetes medications<br>Integration of depression management with diabetes treatment<br>Comparator: usual primary care	12 weeks	Patients in the integrated care group had significantly greater depression remission (PHQ-9 <5: 58.7% vs 30.7%) and larger reductions in depressive symptoms compared with usual care.
Cummings et al. (2019) [14]	Academic family medicine clinic, rural southeastern USA	Adults aged 18-75 with uncontrolled T2D (HbA1c ≥7.0) and comorbid depressive symptoms (PHQ-2 >3) or diabetes-related distress (DDS-2 >3)	Pragmatic RCT; *N*=67 in the intervention group and *N*=72 in control group	Intervention: 16 sessions of individually integrated care model involving tailored cognitive-behavioral therapy plus the American Diabetes Association guideline over 12 months<br>Small Changes Lifestyle Coaching (for low symptoms) or<br>CBT Problem-Solving Therapy (for higher symptoms) + routine medical care + community health worker support<br>Comparator: usual primary care	12 months	Intervention group had significantly greater improvement in depressive symptoms measured by PHQ-9 (−3.39 vs. −0.90; P = 0.01), than usual care.
Ell et al. (2017) [15]	Community safety-net clinics, Los Angeles, USA	Adults with depression (PHQ-9 ≥10) and at least one chronic illness (diabetes and/or heart disease); predominantly low-income Latino patients	RCT; 348 participants randomized (*N*=178 intervention vs *N*= 170 usual care)	Intervention: Promotora-assisted integrated care model ("A Helping Hand"):<br>Bilingual community health workers (promotoras) delivering psychoeducational and problem-solving sessions<br>Six weekly sessions plus three monthly booster sessions<br>Support for depression management, chronic disease self-care, and patient activation<br>Integration with primary care team within a patient-centered medical home model<br>Comparator: Patient-centered medical home usual care	6 months and 12 months	Both groups showed significant improvements in depressive symptoms over time; however, there were no significant differences between intervention and usual care groups. Improvements were likely influenced by system-wide behavioral health integration in the safety-net clinics during the study period.

Izquierdo et al. (2018) [16]	Community service sectors in Los Angeles, California, USA	Adults aged >50 years with depressive symptoms (8-item PHQ score ≥ 10)	Cluster RCT: *N* = 206 in Community Engagement and Planning (CEP) and *N*=188 in Resources for Services (RS)	Intervention: CEP, using a community-partnered multi-sector coalition approach to implement depression collaborative care<br>Comparator: RS, individual program technical assistance	12 months	CEP had a significantly greater reduction in the percentage of participants with poor mental health–related quality of life (effect size: 9.2%; p < .05) and a higher proportion of participants demonstrating improvement in mental health (effect size: 12.8%; p < .05) at 6 months. No significant differences were observed at 12 months.
Yan Wang, Y., et al. (2025) [17]	Community health centers in two counties (urban and rural primary care settings), China	Adults aged 18–85 years with confirmed T2D and depressive symptoms (PHQ-9 ≥ 10); excluded if severe psychiatric illness, suicidal ideation, or enrolled in another trial	Pragmatic cluster-RCT; 8 community health centers; 630 participants (*N*=275 intervention, *N*=355 control)	Intervention: CIC-PDD: Multiprofessional team (diabetes specialist, psychiatrist/psychotherapist, primary-care case managers, student health communicators); structured treatment manual; measurement-based collaborative care; behavioral activation; WeChat-supported follow-up; up to 18 sessions over 12 months; interprofessional case conferences.<br>Comparator: enhanced usual care (PCPs informed of PHQ-9 results; routine diabetes management)	6 and 12 months	At 12 months, 62.1% (intervention) vs. 31.0% (control) achieved ≥50% reduction in depressive symptoms on SCL-20 (risk difference = 31.03%, 95% CI [21.85, 40.21]); mental health–related QoL (SF-12 MCS) improved (difference = 6.74, 95% CI [3.75, 9.74]).
Krahn et al. (2006) [23]	Elderly primary care patients, USA	Elderly primary care patients recruited from ten sites who met the criteria on the MINI for depressive disorders, with or without comorbid anxiety.	Multisite RCT; *N* = 1,531 (*N*= 758 ICM; *N*= 773 specialty referral)	Intervention: PRISM-E:<br>Licensed mental health providers delivered<br>ICM including mental health services, substance abuse services, or both in the primary care clinic.<br>Comparator: enhanced specialty referral model including mental health services, substance abuse services, or both in a specialty setting that is physically separate and designated as a mental health or substance abuse clinic	3 months and 6 months	For major depression, enhanced specialty referral was associated with a significantly greater reduction in depression severity compared with integrated care (Difference = 2.8, 95% CI: 1.0–4.5; p = .003).
Chen et al. (2022) [22]	Rural primary care clinics, China	Clinic patients aged ≥60 years with a diagnosis of hypertension and depressive symptoms (PHQ-9 score ≥10)	Cluster RCT; 218 rural villages, *N*= 1,232 in intervention vs *N* =1,133 n usual care	Intervention: COACH integrated management delivered in rural primary care:<br>Team-enabled approach: village primary care doctors supported by village lay workers with telephone consultation from centrally located psychiatrists.<br>Structured management of both depression and hypertension<br>Comparator: Enhanced care-as-usual	12 months	COACH participants showed greater reduction in depressive symptoms (Cohen's d [±SD] = −1.43 [−1.71, −1.15]; p < 0.001) measured by the HDRS total score; and greater hypertension control
Kvalbein-Olsen et al. (2025) [24]	Primary care centers in Norway	Adults aged ≥65 years with moderate depressive symptoms	Cluster-RCT: *N*=18 in intervention and *N*=16 in control	Intervention: IMPACT: a structured collaborative intervention model involving GPs and geriatric psychiatrists: two consecutive joint consultations with the GP, patient, and geriatric psychiatrist, supplemented by individual GP-patient	12 months	Both groups showed significant reduction in PHQ-9 (3.4 and 4.0, respectively) at 18

		(PHQ-9 scores of 10–19)		consultations. An individual treatment plan was constructed for each patient which could include medication adjustments, municipal healthcare services, social services, as needed.<br>Comparator: regular depression treatment by GP		months, but there was no statistically significant difference between the groups.
Bosanquet et al. (2017) [18]	Primary care practices in UK	Adults aged ≥ 65 years with major depressive disorder	RCT; *N*=485 patients (collaborative care intervention vs usual care)	Collaborative care: behavioral activation, usual general practitioner (GP) care<br>Comparator: usual GP care	4, 12, and 18 months	The collaborative care had a significantly greater reduction in PHQ-9 at 4 months (8.98 vs. 10.90 score points; $p < 0.001$), but the treatment differences were not significant at 12 months or 18 months.
Gellis et al. (2014) [25]	Hospital-affiliated home healthcare agency, USA	Medically frail homebound older adults aged ≥ 65 with heart failure or COPD and comorbid depression (PHQ-2 ≥3); mean age 79 years	RCT; *N*=57I-TEAM; *N*=58 UC+P	Intervention: 3-month integrated telehealth comprising daily telemonitoring (weight, BP, pulse, O2, temperature) + 8 weekly PST sessions (35 min/telephone) + antidepressant prescription + nurse care coordination<br>Comparator: Usual Care + Psychoeducation (UC+P) in-home nursing + weekly 1-hour face-to-face visits + disease education	3 months and 6 months	I-TEAM group had significantly lower PHQ-9 at 3 months (7.4 vs. 13.6; p=.01) and 6 months (7.9 vs. 14.1; p=.05), lower HAM-D scores at 3 months (9.8 vs. 18.6; p=.02) and at 6 months (10.4 vs. 17.4, p=.05), and better SF-12 MCS scores at 3 and 6 months (53.6 vs 42.8, p=.01; and 52.1 vs 40.3, p=.05, respectively).
Janssen et al. (2023) [19]	Primary care centers in Netherlands	Older adults (≥65 years) with depressive symptoms (PHQ-9 ≥ 10)	Cluster RCT: *N*=96 for behavioral activation and *N*= 65 for treatment as usual	Intervention: behavioral activation, with trained mental health nurses to deliver an 8-week behavioral activation protocol<br>Comparator: treatment as usual	12 months	The behavioral activation participants had significant reduction in functional limitations at the end of treatment (WHODAS 2.0 difference −3.62, p = 0.01), but the difference was not significant at the 12-month follow-up
Seward et al, (2025) [26]	Primary care clinics in Brazil	Adults aged 60 years or older with depressive symptomatology (PHQ-9 score ≥10)	Clustered RCT: *N*=360 in the intervention arm and *N*=355 in the control arm	Intervention: PROACTIVE, a task-shared, stepped and collaborative care model that transferred selected specialist clinical responsibilities to trained and supervised community health workers. The intervention included psychoeducation and behavioral activation. It adopted a stepped-care approach, allocating more intensive resources to participants with severe conditions, while those with milder symptoms received lower-intensity support.<br>Comparator: enhanced usual care	12 months	The intervention group had a significantly higher recovery from depression (PHQ-9 score <10) at the 8-month follow-up assessment (62.5% vs 44.0%; odds ratio =2.16, p<0.0001) and at the 12-month follow-up assessment (odds ratio= 2.33, p<0.0001)
Liao et al. (2022) [27]	Community-dwelling older adults,	Adults with a medical diagnosis of depressive disorder or depressive mood,	Quasi-randomized study; *N* = 67 in intervention and *N*=76 in control	Intervention: trained nurses to deliver ICM intervention:<br>Assessment and managing of health problems<br>Achieving mental and spiritual wellbeing,	12 weeks and 18 weeks	Greater reduction in depression scores (CES-D) in the intervention group (ICM) at 18

	Taiwan	BSRS-5 > 5; MMSE > 20		Enhancing activities of daily living,<br>Providing social welfare services and prevention of elder abuse<br>Comparator: usual care		weeks (β = −4.76, 95% CI: −7.39 to −2.14, p < .001)
Wang, N., et al. (2024) [28]	Urban and suburban community health centers, Beijing China	Community-dwelling older adults at risk of functional (intrinsic capacity) decline; screened via ICOPE Step 1	RCT; *N*=537 intervention, *N*=1,611 control; 938 matched pairs after propensity score matching	Intervention: WHO ICOPE program localized to China; holistic assessment across five intrinsic capacity domains including psychological health; individualized care plans; care-manager coordination; proactive community-based follow-up.<br>Comparator: standard community care	6 months	Psychological health (GDS-5) significantly improved in the intervention group vs. control (LS mean difference 0.09, 95% CI [0.03, 0.14], p < .05)
Almeida et al, (2021) [20]	Community residents in rural Western Australia	Adults aged 65 years and above with positive response to either of the Whooley questions	RCT, *N*= 154 in behavioral activation and *N*=153 in usual care	Intervention: usual care plus a phone-delivered behavioral activation program consisted of a collaborative care, self-managed behavioral activation program supported by a trained psychologist over the phone for 8 weeks.<br>Comparator: usual care	52 weeks	Participants in the intervention group experienced significant improvements in depression and anxiety over 52 weeks: mean difference in PHQ-9 scores = 1.0 (p = 0.048), difference in GAD-7 =1.0 (p = 0.013)
Ng et al., (2020) [21]	Community-based outreach service centers in Singapore	Adults aged 60 or older with depressive symptoms (15-items Geriatric Depression Scale [GDS] ≥5)	RCT: *N* = 102 in collaborative care and *N* = 112 in usual care	Intervention: Collaborative care delivered by trained general practitioners (GPs), following a care management protocol that includes antidepressant treatment, referral to mental health specialists, and case management over a six-month period<br>Comparator: usual care	12 months	Participants in the collaborative care group showed a significantly greater reduction in HDRS scores compared to the usual care group at 6 months (3.5 vs. 2.0). At 12 months, both groups showed similar levels of maximal improvement.
Liu et al. (2026) [29]	Community setting in Hong Kong	Adults age ≥60 years, at risk of depression (PHQ-9 < 5) and having risk factors, or having mild to moderately severe depressive symptoms (PHQ-9 of 5-19)	Pragmatic quasi-experimental trial: JoyAge (*N* = 2,975) and usual care (*N* = 441).	Intervention: "JoyAge", a collaboration between aged care and mental health care units, evidence-based psychosocial interventions delivered by trained social workers, peer support, and stepped care according to depressive symptom level.<br>Referral to specialist mental health services for severe cases<br>Comparator: care as usual (CAU)	2-12 months	JoyAge participants showed greater reduction in PHQ-9 (adjusted mean difference [AMD]= 1.65, p<0.001) anxiety (AMD = 1.47, p < .001), and loneliness (AMD = 1.29, p < .001)

Abbreviations:

BSRS: Brief Symptom Rating Scale; CBT: Cognitive Behavioral Training; COACH: Chinese Older Adult Collaborations in Health; DDS: Diabetes Distress Scale; ED: emergency department; HAM-D: Hamilton Depression Rating Scale; HDRS: Hamilton Depression Rating Scale; ICM: Integrated care model; I-TEAM: Integrated Telehealth Education and Activation of Mood; MCS: Mental Component subscales; MINI: Mini-International Neuropsychiatric

Interview; MMSE: Mini-Mental State Examination; PHQ: Patient Health Questionnaire; PST: Problem-solving treatment; RCT: randomized controlled trial; SF: Short-Form Survey; T2D: type 2 diabetes; UC+P: Usual in-home care plus psychoeducation; WHODAS: World Health Organization Disability Assessment Schedule
